# A Biodegradable Antifungal-Loaded Sol–Gel Coating for the Prevention and Local Treatment of Yeast Prosthetic-Joint Infections

**DOI:** 10.3390/ma13143144

**Published:** 2020-07-15

**Authors:** David Romera, Beatriz Toirac, John-Jairo Aguilera-Correa, Amaya García-Casas, Aránzazu Mediero, Antonia Jiménez-Morales, Jaime Esteban

**Affiliations:** 1 Clinical Microbiology Department, IIS-Fundación Jiménez Díaz, UAM, 28040 Madrid, Spain; jesteban@fjd.es; 2 Materials Science and Engineering Department, University Carlos III of Madrid, 28911 Madrid, Spain; btoirac@ing.uc3m.es (B.T.); amayagarciacasas@gmail.com (A.G.-C.); toni@ing.uc3m.es (A.J.-M.); 3 Bone and Joint Unit, IIS-Fundación Jiménez Díaz, UAM, 28040 Madrid, Spain; aranzazu.mediero@quironsalud.es

**Keywords:** *Candida*, fungal prosthetic-joint infection, osteointegration, sol–gel coating, antifungal, anidulafungin

## Abstract

Fungal prosthetic-joint infections are rare but devastating complications following arthroplasty. These infections are highly recurrent and expose the patient to the development of candidemia, which has high mortality rates. Patients with this condition are often immunocompromised and present several comorbidities, and thus pose a challenge for diagnosis and treatment. The most frequently isolated organisms in these infections are *Candida albicans* and *Candida parapsilosis*, pathogens that initiate the infection by developing a biofilm on the implant surface. In this study, a novel hybrid organo–inorganic sol–gel coating was developed from a mixture of organopolysiloxanes and organophosphite, to which different concentrations of fluconazole or anidulafungin were added. Then, the capacity of these coatings to prevent biofilm formation and treat mature biofilms produced by reference and clinical strains of *C. albicans* and *C. Parapsilosis* was evaluated. Anidulafungin-loaded sol–gel coatings were more effective in preventing *C. albicans* biofilm formation, while fluconazole-loaded sol–gel prevented *C. parapsilosis* biofilm formation more effectively. Treatment with unloaded sol–gel was sufficient to reduce *C. albicans* biofilms, and the sol–gels loaded with fluconazole or anidulafungin slightly enhanced this effect. In contrast, unloaded coatings stimulated *C. parapsilosis* biofilm formation, and loading with fluconazole reduced these biofilms by up to 99%. In conclusion, these coatings represent a novel therapeutic approach with potential clinical use to prevent and treat fungal prosthetic-joint infections.

## 1. Introduction

Prosthetic-joint infections (PJIs) are a highly debilitating complication affecting the joint prosthesis and adjacent tissue that occurs in approximately 1–3% of patients who undergo total arthroplasty [1,2]. The majority of these infections are caused by bacteria, while only 1% are caused by fungi [3]. In particular, fungal PJI are highly persistent and recurrent, exposing patients to the development of candidemia, a severe complication with a high rate of mortality (30–60%) [4]. These infections are mostly caused by *Candida* species, being *Candida albicans* (C.P. Robin) Berkhout and *Candida parapsilosis* Langeron & Talice the most frequently isolated species [5,6] and other filamentous fungi such as *Coccidioides immitis* C.W. Stiles [7] or *Aspergillus* spp. [8], and in a small percentage of cases there may be a concomitant bacterial infection [3]. When the fungus encounters the implant surface, it develops a complex biofilm which displays potent resistance to antimicrobial therapy and protects the community of microbes from the immune response, which is the cause for the high persistence of the infection [9].

Moreover, patients who develop these infections usually have different clinical characteristics than those with bacterial PJI, and they use to present pharmacological or innate immunosuppression, prolonged or inappropriate use of antibiotics, and the presence of comorbidities including cardiac, renal, or liver diseases [10,11]. Immunosuppression is considered the most important risk factor for developing a recurrent infection in these patients, as elements of innate immunity are the first to interact with the fungus, thus making them essential to the induction of specific secondary responses [12].

Fungal PJI often require complex management and treatment, including aggressive debridement to clean and disinfect the affected area as well as long-term courses of antifungal therapy [3,7], thus substantially increasing economic costs. Currently, two-stage exchange arthroplasty is considered the best treatment option, with success rates ranging from 50% to over 90% [13,14]. In a significant percentage of patients, however, final reimplantation cannot be completed due to the poor health of the patient or persistent infection, which increases the risk of therapeutic failure and mortality [15,16].

To date, several strategies devised to prevent and treat implant infections involve the use of antimicrobial-loaded spacers, surface modification of the biomaterial, or drug-delivery systems to combat the infection locally [17,18]. However, most of these are focused on bacterial PJIs, and scant attention has been paid to infections resulting from the presence of fungi. In recent years, an increase has been seen in the incidence of fungal PJIs, mainly due to inappropriate use of broad-spectrum antimicrobials [2,19]. In consequence, there is a need for effective prevention and treatment strategies due to the drawbacks of existing approaches. By way of example, acrylic antifungal-loaded cement spacers are currently a valuable technique for the treatment of fungal PJIs, although there exists concern about the appropriate quantity of antifungal agent to be loaded; furthermore, cements do not promote osseointegration, and as a result the risk of reinfection is not minimized [20]. Despite these limitations, recent works in this field of research have successfully tested the use of zinc implants that promote osseointegration and display anti-biofilm capacities in vitro against *C. albicans* [21], irreversible immobilization of antifungal drugs on the biomaterial surface [22], and polymer hydrogels with adsorbed antifungal drugs [23].

In previous publications, the osseointegration capacity of a hybrid organo–inorganic biodegradable sol–gel coating biofunctionalized with phosphorous compounds was demonstrated. These coatings significantly increased the proliferation of mouse osteoblasts (MC3T3-E1 cells) and induced matrix mineralization through the release of phosphate groups during sol–gel degradation in aqueous solution within the first 24 to 48 h, i.e., the critical period for the development of an implant infection [24]. This would promote a distance osseointegration of the implant, i.e., a newly formed peri-implant bone trabeculae that develop from the host bone cavity towards the implant surface [25]. Furthermore, the coatings were shown to be almost hydrophobic, showing a water contact angle = 87.5°, which negatively affects bacterial adhesion [26] and makes these coatings a promising tool for the prevention of bacterial implant-related infections. In a subsequent work using in vitro and in vivo experiments, the addition of moxifloxacin to the coatings proved to effectively prevent bacterial implant infections [27]. In another work, fluconazole and anidulafungin-loaded coatings without the addition of phosphorus compounds were characterized electrochemically and the release of both antifungals was evaluated [28].

The present study is focused on the microbiological evaluation of these coatings biofunctionalized with organophosphite and different concentrations of fluconazole and anidulafungin to prevent and/or treat locally implant-related infections caused by fungi. The use of the sol–gel technology provides a simple, rapid and affordable tool that does not require neither the use of high temperatures nor the pretreatment of the biomaterial surface during the process, which increases its versatility by allowing the incorporation of functional biomolecules [29]. Since no similar biomaterials are currently available, the use of these coatings is a highly novel and promising approach to combat these infections.

## 2. Materials and Methods

### 2.1. Preparation of Sol–Gel Coatings and Substrates

Hybrid organo–inorganic sol–gel coatings composed by a mixture of organopolysyloxanes: methacryloxypropyltrimethoxy silane (MAPTMS, 98%, Acros Organics, Thermo Fisher Scientific, Waltham, MA, USA) and tetramethyl orthosilane (TMOS, 98%, Acros Organics, Thermo Fisher Scientific, Waltham, MA, USA) and biofunctionalized with tris(tri-methylsilyl)phosphite (92%, Sigma–Aldrich, St. Louis, MO, USA) were prepared following a previously published methodology [24]. Three coatings were loaded with the following quantities of fluconazole (Sigma-Aldrich, St. Louis, MO, USA): 0.65 (F50), 0.975 (F75), and 1.3 (F100) mg/mL sol–gel; alternatively, they were loaded with anidulafungin (Pfizer, New York, NY, USA) in the following quantities: 0.49 (A50), 0.74 (A75), and 0.99 (A100) mg/mL sol–gel. Functionalization of the coatings with antifungal drugs was performed by adding the drug to the aqueous phase during its preparation. These concentrations have been intentionally chosen because they represent 50%, 75% and maximum amount of this antifungal which sol–gel can contain without compromising its stability, durability, and adherence on titanium (Ti) substrates. Sol–gel functionalized with organophosphite without the addition of antifungals was used as a control (P2).

### 2.2. Surface Characterization

The surface morphology and composition of the as-prepared coatings were assessed by scanning electron microscopy (SEM) and Energy Dispersive Spectrometry (EDS). The homogeneity of applied coatings was analyzed. The study was performed using the Teneo FEI Tungsten filament Electronic Microscope (Field Electron and Ion Company, FEI, OR, USA), equipped with an X-ray microanalysis system along with an Octane Plus detector of 30 mm^2^ area. Images were taken applying 2 kV and 0.2 nA at 1000× magnifications and the Circular Back-Scatter detector (CBS) was used.

### 2.3. Kinetics Study of Antifungal Release

These experiments were performed using coatings F100 and A100. Briefly, release analyses of both antifungals were based on multiple absorbance measurements using a JASCO V-650 UV–vis absorption spectrophotometer (Jasco Deutchsland Gmbh, Pfungstadt, Germany). Coatings were exposed to 5 mL of Dulbecco’s Phosphate Buffered Saline (PBS) solution (pH 7.4) (Sigma–Aldrich, St. Louis, MO, USA) at 37 °C and placed in polypropylene tubes. Three samples were used for each coating for the study. The fluconazole and anidulafungin release were monitored by measuring the maximum absorbance of both antifungals (261 nm and 303 nm respectively) at different times (2, 4, 6, 12, 24, and 48 h). For each measurement, 3 mL aliquots were extracted and transferred to a 3 mL quartz cuvette (10 mm path length, Hellma GmbH, Müllheim, Germany). The drug concentration for the corresponding absorbance values was calculated based on the calibration curves for fluconazole and anidulafungin in PBS, previously made. Calibration was performed by varying the concentration of both antifungals between 0.1 × 10^−3^ mg to 0.1 mg. Then, the concentration of each sample was normalized considering the dilution and the corresponding calibration curve. The calibration curves were linear in the concentration range measured with an R^2^ = 0.9999 for the fluconazole calibration curve and R^2^ = 0.9990 for the anidulafungin calibration curve.

### 2.4. Selection and Maintenance of Strains for Microbiological Study

Experiments were performed using the reference strain *C. albicans* from the American Type Culture Collection ATCC 10,231 in addition to two *C. albicans* clinical isolates: Cal1 (isolated from a catheter infection) and Cal35 (isolated from a hip PJI); and reference strain *C. parapsilosis* ATCC 22,019 plus two clinical isolates: κ1 (isolated from a case of otitis) and κ4 (isolated from a hip PJI). Clinical isolates were identified by MALDI-TOF-TOF using the Vitek MS system (database IVD V3.0.) (BioMérieux, Marcy-l’Étoile, France). All strains were kept frozen at −80 °C until the experiments were performed. They were then maintained at 37 °C in Sabouraud gentamicin chloramphenicol agar plates (SGC2) (BioMérieux, Marcy-l’Étoile, France).

### 2.5. Adherence Study

To evaluate the adherence of the strains to the sol–gel without the addition of antifungals, the bottom of a well of a six-well polystyrene plate (Thermo Fisher Scientific, Waltham, MA, USA) was coated with 100 μL of sol–gel (P2) and cured at room temperature for at least 24 h. Next, 3 mL of a solution diluted to a final concentration of 0.5 McFarland (0.5–2.5 × 10^5^ Colony forming units (CFU)/mL) of yeast in sterile saline solution (SS) was added to each well and the plate was incubated for 2 h at 37 °C and 5% CO_2_. After incubation, the wells were washed two times with 2 mL of SS, and another 2 mL of SS were added to the well, after which the plate was sonicated for 5 min at 50 to 60 Hz. Following sonication, the yeasts that had adhered to the coating were estimated by means of the drop plate method [30]. As a positive control, the experiment was performed with uncoated wells.

### 2.6. Evaluation of Biofilm Formation Inhibition

To evaluate the inhibition of biofilm formation, all sol-gel formulations were deposited on Ti sample pieces of 15 mm diameter × 25 mm thick prepared by a conventional powder metallurgy route by dip-coating as described previously [24]. Then, the coatings were dried at 60 °C for one hour inside an oven. The coated Ti substrates were placed in a well of a 12-well plate (Sigma Aldrich, St. Louis, MO, USA) with 3 mL of a solution diluted to a final concentration of 0.5 McFarland of yeasts in Roswell Park Memorial Institute (RPMI) 1640 medium (Thermo Fisher Scientific, Waltham, MA, USA) supplemented with glucose 2% and buffered with 3-(N-morpholino) propanesulfonic acid (MOPS) (Sigma Aldrich, St. Louis, MO, USA) 0.165 mol/L at pH 7.0, and incubated at 37 °C in 5% CO_2_ for 24 h in the case of *C. albicans* strains [31] and for 48 h in the case of *C: parapsilosis* strains [32]. After incubation, the Ti pieces were washed three times in SS and sol–gel coatings were scraped using sterile wooden sticks that were then sonicated in 10 mL of SS. Then, CFU/cm^2^ values were estimated by the drop plate method. Additionally, non-adherent planktonic yeasts remaining in the incubation medium were estimated by absorbance at 530 nm, using RPMI medium alone as a negative control.

### 2.7. Evaluation of the Treatment of Mature Biofilms

Biofilm formation was induced by inoculating 200 µL/well of a solution diluted to a final concentration of 0.5 McFarland of yeasts in RPMI 1640 + 2% glucose + MOPS on untreated, flat-bottomed, 96-well Fluoronunc Black polystyrene microtiter plates (Thermo Fisher Scientific, Waltham, MA, USA) incubated for 48 h at 37 °C and 5% CO_2_. After incubation, the medium was removed and 200 µL of fresh media was added to each well, and the lid of the plate was replaced by a MBEC™ biofilm Incubator lid (Innovotech, Edmonton, AB, Canada); the previous day, the lid pegs had been coated by dipping the lid in wells filled with 200 µL of each sol–gel formulation (negative control, P2, F/A50, F/A75, and F/A100; *n* = 8 for each) followed by incubation at 37 °C in 5% CO_2_ for 48 h. Biofilm viability was then determined by adding 10 µL of AlamarBlue^®^ (BIO-RAD, Hercules, CA, USA) to each well and incubating the plate with gentle shaking (70 rpm) for 3 h at 37 °C. Fluorescence was measured in a Perkin Elmer EnSpire^®^ Multimode Reader (Perkin Elmer, Waltham, MA, USA) using an excitation wavelength of 570 nm and an emission wavelength of 585 nm. Fresh RPMI 1640 medium alone was used as a negative control and biofilms grown alone were used as positive controls.

### 2.8. Cellular Study

MC3T3-E1 cells were seeded at a concentration of 10,000 cells/cm^2^ on 96-well plates with α-minimum essential medium with 10% bovine fetal serum and 1% penicillin-streptomycin (αMEM, Invitrogen, Thermo Fisher Scientific, Waltham, MA, USA) and were incubated at 37 °C and 5% CO_2_ overnight. After cell adherence, the medium was replaced by αMEM with 50 mg/mL ascorbic acid (Sigma–Aldrich, St. Louis, MO, USA), 10 mM ß-glycerol-2-phosphate (Sigma–Aldrich, St. Louis, MO, USA) to promote osteoblastic differentiation, and the lid of the plate was replaced with a MBEC™ biofilm incubator lid (Innovotech, Edmonton, AB, Canada); the previous day, the lid pegs had been coated by dipping the lid in wells filled with 200 µL of each sol–gel formulation (negative control, P2, F/A50, F/A75, and F/A100; *n* = 8 for each) followed by incubation at 37 °C in 5% CO_2_ for 48 h. After incubation, cytotoxicity was tested by CytoTox 96^®^ NonRadioactive Cytotoxicity Assay (Promega, Madison, WI, USA). Cell proliferation was determined by addition of AlamarBlue^®^ solution (BIO-RAD, Hercules, CA, USA) at 10% (v/v) to the cell culture at 48 h of growth. Fluorescence intensity was measured with excitation and emission wavelengths of 540 and 600 nm, respectively, in a Tecan Infinite 200 Reader (Tecan Group Ltd., Männedorf, Switzerland).

### 2.9. Statistical Analysis

All statistical analyses were performed using the Stata software program, release 11 (Statacorp, College Station, TX, USA). First, the normality of each series of data was checked with the Shapiro–Wilk test. Results from the drug-release experiments were analyzed by using the non-parametric Kruskal–Wallis test. Results from the adherence study and the evaluation of the inhibition of biofilm formation were analyzed by using the Wilcoxon test to compare each group with the control. The analysis of the percentage of biofilm viability and the cellular study were performed using Student’s t-test to compare each group with controls. For analyzing the antifungal release, the data were adjusted to a linear regression model and a non-parametric Kruskal–Wallis test was employed to compare the drug release between the different times. A level of significance of 0.05 was used in all tests. All data are represented as mean and standard deviation for statistically normal results and as median and interquartile range for non-normal results. All experiments were performed in at least three biological replicates.

## 3. Results

### 3.1. Synthesis of the Coatings and Surface Characterization

**Figure 1:**
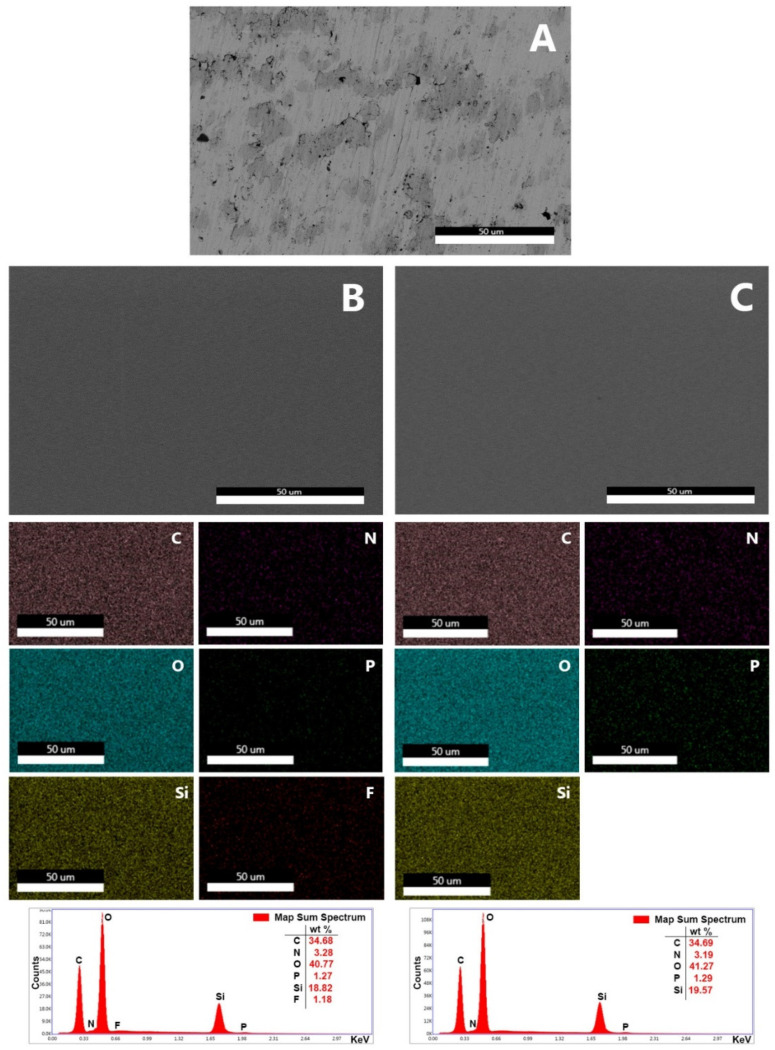
CBS-SEM micrographs of the Ti-powder metallurgy substrate (**A**), F100 (**B**) and A100 (**C**) surfaces. Energy dispersive spectroscopy (EDS) elemental mapping images and EDS spectrum of F100 (**B**) and A100 (**C**) coatings showing the distribution of chemical elements. C: Carbon; N: Nitrogen, O: Oxygen; P: Phosphorus; Si: Silicon; F: Fluor.

The obtained sol in all cases was transparent and without phases separation. Sols had an adequate viscosity facilitating the correct application on the substrate. Dried coatings were simple-sight observed, without imperfections such as cracks or pores. A more thorough inspection was performed using SEM. Figure 1 shows SEM images observed with CBS detector to study the composition of the substrate and the coatings F100 and A100 applied onto the titanium substrate. In addition, the figure shows an element mapping using EDS to study the distribution of the elements. Inspection of the surfaces showed the formation of smooth, uniform, homogeneous, and crack-free coatings on the substrates in these formulations.

### 3.2. Drug-Release Study

**Figure 2:**
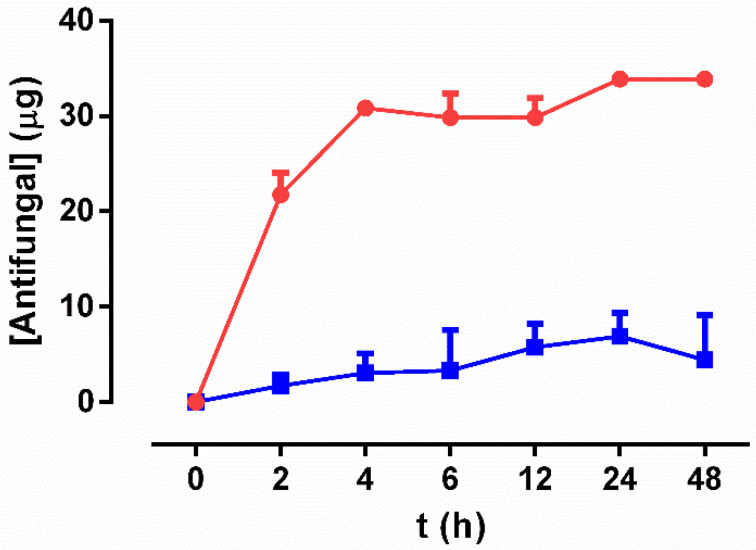
Kinetics of fluconazole (**red**) and anidulafungin (**blue**) released from F100 and A100 sol–gels over time. Data represent median and interquartile range of the amount of drug (in µg) measured in three replicates.

The release of fluconazole followed a linear behavior between 0 and 4 h (R^2^ = 0.9116) with a release constant of 7.44 µg/h, and reaching a concentration of 30 μg at 4 h. From 6 to 48 h the release of fluconazole stabilized and stayed constant over time (*p* = 0.478 for Kruskal–Wallis test). In contrast, anidulafungin release followed a similar pattern, most of the drug was released within the first 4 h (R^2^ = 0.7286) with a release constant of 0.899 µg/h and reaching a concentration of up to 7 µg, staying constant from 6 to 48 h (*p* = 0.478 for Kruskal–Wallis test) (Figure 2).

### 3.3. Adherence Study

**Figure 3:**
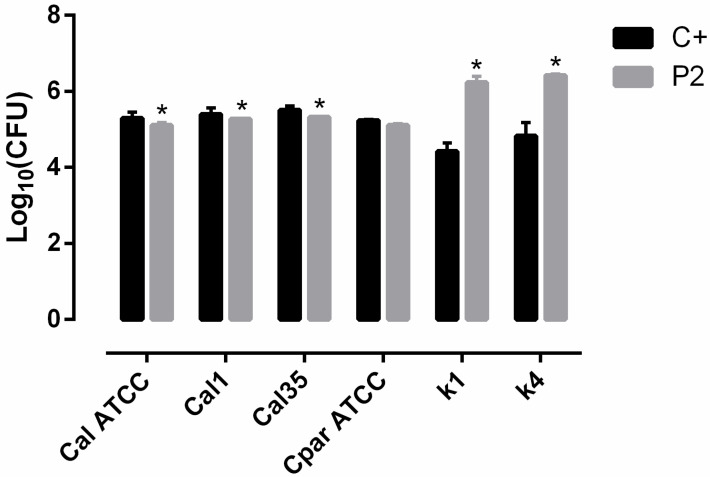
Adherence study of yeasts attached to the uncoated (C+) or coated (P2) bottom of a P6 well plate. Data are represented as log_10_(median of CFU) estimated in three biological replicates *: *p* < 0.05 for Wilcoxon test between the two compared conditions. The bars represent median and interquartile range of log_10_(CFU) estimated in three biological replicates. Cal ATCC: *C. albicans* ATCC 10231. Cpar ATCC: *C. parapsilosis* ATCC 22019.

The presence of the sol–gel coating (P2) slightly decreased the adhesion of *C. albicans* strains (*p* = 0.0495). In contrast, there was no significant effect on *C. Parapsilosis* (*p* = 0.1266), and P2 promoted adherence of *C. parapsilosis* clinical strains 39 to 66-fold for κ1 and κ2, respectively, in comparison with the uncoated control (*p* = 0.04) (Figure 3).

### 3.4. Prevention of Biofilm Formation

**Figure 4:**
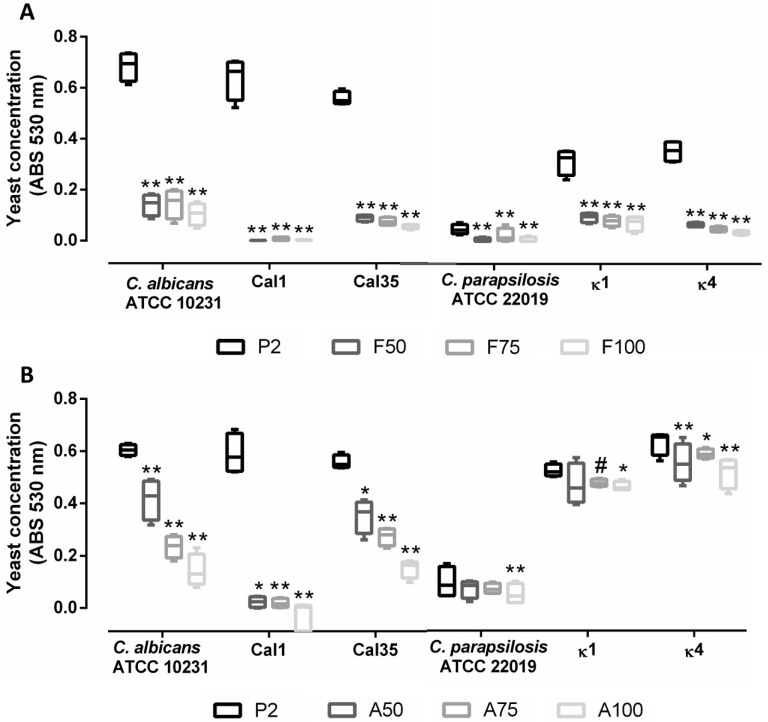
Quantification of non-adherent planktonic yeasts following incubation of titanium pieces coated with P2 (control), fluconazole-loaded coatings (**A**) or anidulafungin-loaded coatings (**B**). Data are represented as median and interquartile range of absorbances (absorbance units, AU) obtained in at least three independent experiments. *: 0.05 > *p* > 0.01; **: *p* < 0.01, #: 0.051 > *p* > 0.099 for Wilcoxon test between the control coating (P2) and the other coatings.

An evaluation of non-adherent yeasts showed that loading with fluconazole was effective in reducing the generation of planktonic yeasts by up to 75% in all strains (Figure 4A). The load with anidulafungin showed a marked concentration-dependent effect in *C. albicans* strains, though the same was not observed with *C. parapsilosis* strains. In both cases, A100 produced the highest reduction of planktonic yeasts (Figure 4B). Comparing the two antifungals, fluconazole caused a higher reduction in planktonic yeasts than anidulafungin, especially in *C. parapsilosis* strains. Anidulafungin did not produce a significant reduction in yeasts in *C. parapsilosis* strains.

**Figure 5:**
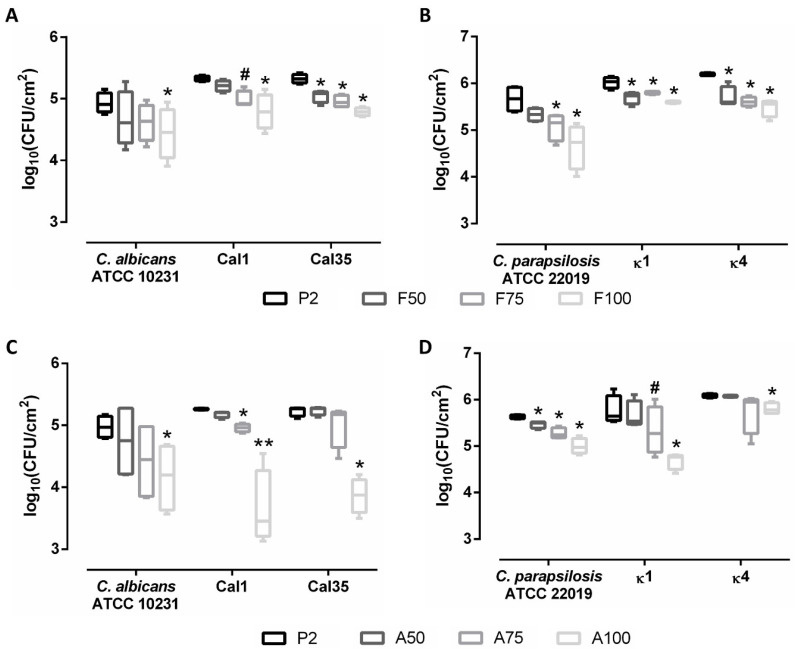
Quantification of biofilms formed on titanium pieces coated with P2 (control), fluconazole-loaded coatings (**A**,**B**), or anidulafungin-loaded coatings (**C**,**D**). Data are represented as median and interquartile range of the log_10_(CFU/cm^2^) estimated by drop plate in at least three independent experiments. *: 0.05 > *p* > 0.01; **: *p* < 0.01; #: 0.051 > *p* > 0.099 for Wilcoxon test between the control coating (P2) and the other coatings.

When evaluating the prevention of biofilm formation, antifungal loading showed a concentration-dependent effect: coatings loaded with maximum concentrations of fluconazole (F100) or anidulafungin (A100) caused the greatest inhibition in biofilm development in both species, inhibiting both species by up to 60% (Figure 5). Moreover, loading with anidulafungin prevented biofilm formation more efficiently than fluconazole in *C. albicans* strains (Figure 5C), while the latter was more efficient against *C. parapsilosis* strains (Figure 5B).

### 3.5. Evaluation of Treatment of Mature Biofilms

**Figure 6:**
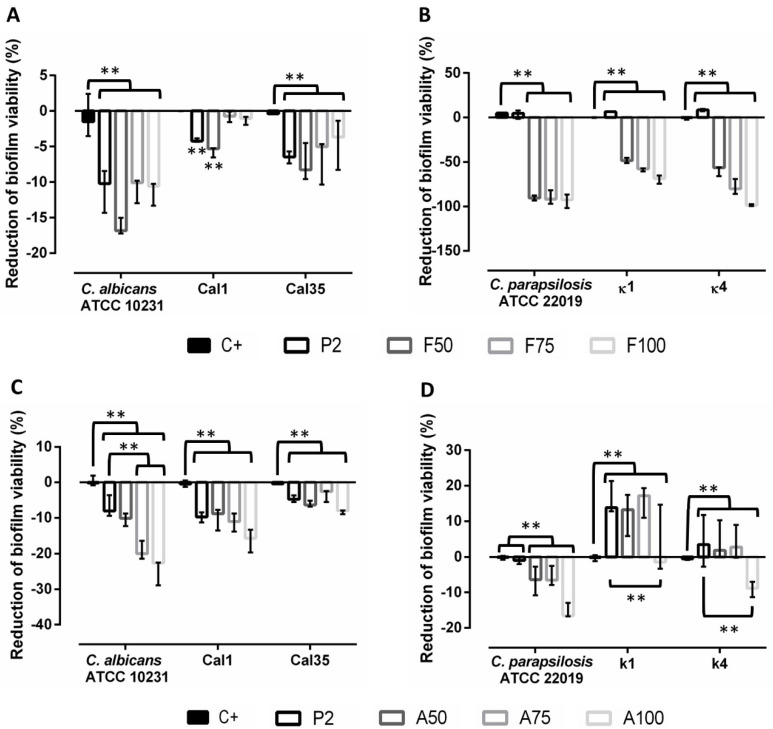
Percentage of reduction of biofilms treated with fluconazole-loaded coatings (**A**,**B**) or anidulafungin-loaded coatings (**C**,**D**). Comparisons with respect to positive control (without treatment) and P2. Data are represented as mean and standard deviation of the percentage of viability reduction estimated in at least three independent experiments. *: 0.05 > *p* > 0.01; **: *p* < 0.01 for *t*-test.

The results of this experiment showed that the presence of the unloaded coating (P2) was sufficient to produce a significant decrease in biofilm viability of *C. albicans* strains (*p* < 0.001), and the addition of antifungals contributed synergistically to this effect (Figure 6A). The addition of anidulafungin only had a significant effect on *C. albicans* reference strain, producing a greater decrease in biofilm viability compared to P2. This effect was not significant in clinical strains, although no concentration-dependent trend was observed (Figure 6C).

In contrast, P2 induced slight biofilm production in *C. parapsilosis* strains, and the presence of antifungals had a differential effect. Fluconazole was much more effective than anidulafungin, reducing biofilm formation by up to 99% in *C. parapsilosis* reference and κ4 strains (Figure 6B,D).

### 3.6. Cytotoxicity and Proliferation Assays

**Figure 7:**
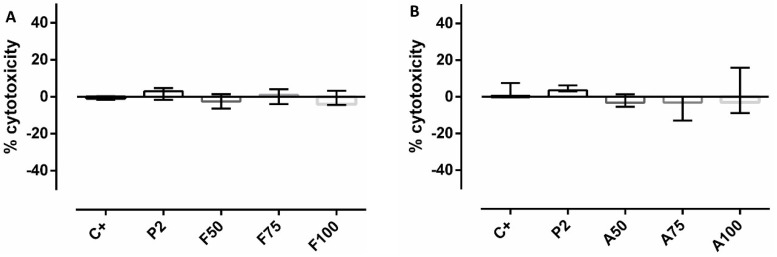
Percentage of MC3T3-E1 cytotoxicity without treatment (positive control, C+) or treated with P2 or with the fluconazole-loaded (**A**) or anidulafungin-loaded (**B**) sol–gel coatings. Bars represent mean and standard deviation of the percentage of cytotoxicity estimated in three biological replicates.

**Figure 8:**
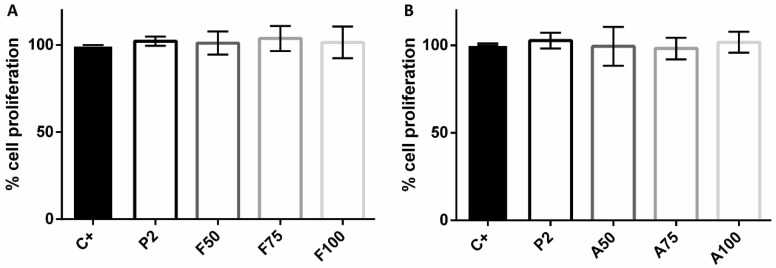
Percentage of MC3T3-E1 proliferation without treatment (positive control, C+), or treated with P2 or with the fluconazole-loaded (**A**) or anidulafungin-loaded (**B**) sol–gel coatings. Bars represent mean and standard deviation of the percentage of cellular proliferation estimated in three biological replicates.

No significant effects on cytotoxicity or cellular proliferation due to the presence of coatings were observed (*p* > 0.05 in all cases) (Figure 7 and Figure 8).

## 4. Discussion

In this work, the research group describes the capacities of a hybrid sol–gel coating loaded with different concentrations of fluconazole or anidulafungin to prevent and/or treat fungal PJI.

First, adhesion experiments showed that P2 reduced *C. albicans* yeast adhesion and promoted the adherence of *C. parapsilosis* yeasts by up to 65-fold. Since this effect is the result of the hydrophobicity of P2, which facilitates the adherence of yeasts through London–van der Waals forces (commonly designated as hydrophobic interaction) [33], the chemistry of the sol–gel degradation could be the cause of the lower adhesion of *C. albicans*. When placed in aqueous solution, water hydrates the net of the sol–gel, swelling the coating before hydrolytic degradation begins. Hydrolytic degradation of the coatings is based on a depolymerization reaction that can be considered the opposite reaction of polycondensation [24]; hence, yeast attachment could be affected as the surface is being continually remodeled on a nanometric scale. In addition, several factors could contribute to the observed differences between both species. For instance, *C. parapsilosis* displays higher surface hydrophobicity than *C. albicans*, thus increasing its ability to adhere to hydrophobic surfaces [34,35]. Moreover, it has been reported that *C. parapsilosis* strains display high genomic variability, with a high percentage of them having numerous repetitions of orthologous genes to *C. albicans* Agglutinin-like sequence (ALS) genes. ALS genes encode adhesins, which are responsible for the initial adhesion of yeasts to biotic and abiotic surfaces [36]. In contrast, *C. albicans* strains maintain a lower and stable copy number of ALS genes [37].

Second, antifungal-loaded coatings effectively prevented biofilm formation of both species in a concentration-dependent manner: A100 was more effective against *C. albicans* strains while F100 was more effective against *C. parapsilosis* strains. The higher tolerance of *C. parapsilosis* strains to echinocandins is well characterized and is due to a sequence variation present in the hot-spot region 1 of the glucan synthase, which decreases its drug sensitivity [38]. Moreover, the evaluation of non-adhered yeasts showed that F100 was the most effective formulation against both species, reducing planktonic yeasts by more than 75% in all strains. This effect could be due to a lower release of the anidulafungin from the molecular framework of sol–gel during hydrolytic degradation due to the larger size of the drug molecules. The hypothesis was confirmed by evaluating the drug release from the coatings after introducing them in aqueous solution. The results showed that the release of fluconazole was much more efficient, with a release constant of 7.44 µg/h within the first 4 h, while anidulafungin showed a release constant of 0.899 µg/h. According to the distribution of Minimum Inhibitory Concentrations published for *C. albicans* and *C. parapsilosis* [39], the amount of fluconazole released would be sufficient to inhibit the growth of *C. albicans* and *C. parapsilosis*, while the amount of anidulafungin released would be effective in inhibiting *C. albicans* but not *C. parapsilosis*, which is in concordance with the results obtained in this work.

In addition, taking the results from the analysis of planktonic yeasts and the inhibition of biofilm formation, the higher adherence of *C. parapsilosis* strains can also be observed indirectly: the estimation of non-adherent *C. albicans* yeasts showed higher absorbance than *C. parapsilosis* yeasts (0.6 versus 0.5), while *C. parapsilosis* CFU/cm^2^ counts were between 5 and 7.5-fold higher than *C. albicans* counts.

Third, biofilm treatment studies showed that the presence of the unloaded coating decreased biofilm viability in *C. albicans* strains, and the addition of antifungals contributed to this effect but was not significant in clinical strains. In contrast, in *C. parapsilosis* strains, the presence of P2 significantly induced biofilm formation, and loading with fluconazole dramatically reduced biofilm viability, while loading with anidulafungin was not effective. This effect may be related to the hydrolytic degradation of the coatings. During sol–gel hydrolytic degradation, phosphate ions are released to media, which are virulence and morphogenesis modulating factors in some yeasts such as *C. albicans* and *C. glabrata* [40,41].

Fourth, higher values of absorbance, CFU/cm^2^ and biofilm formation were obtained in clinical strains regardless of species, consistent with the assumption that clinical strains display a greater capacity to form biofilms than reference strains [42]. This remarks the importance of adding clinical strains to this type of studies, as they tend to behave differently from reference strains in terms of biofilm production capacity and antimicrobial resistance mechanisms.

Last, no significant effects were found on cytotoxicity and proliferation. This is in contrast with previous works where fluconazole-loaded coatings displayed slight cytotoxicity [28]. However, those coatings were not functionalized with phosphorous compounds. Taking into account that the presence of organophosphate enhances cellular proliferation [24,43], it could be overcoming the slight cytotoxicity of fluconazole, which explains the absence of cytotoxicity of the fluconazole-loaded coatings observed in this work.

## 5. Conclusions

The coatings loaded with the highest concentration of antifungals showed an excellent anti-biofilm behavior. Therefore, these coatings could be a useful tool for preventing and treating locally yeast PJI, In particular, the coatings loaded with fluconazole proved to be effective against both *Candida* species tested, nevertheless, and thanks to the versatility that offers the sol–gel technology, other drugs and combinations could be tested, aiming for a more personalized treatment.
